# Gender-inclusive consumer studies improve cassava breeding in Nigeria

**DOI:** 10.3389/fsoc.2024.1224504

**Published:** 2024-02-12

**Authors:** Tessy Madu, Samuel Onwuka, Solomon Nwafor, Mercy Ejechi, Miriam Ofoeze, Nnaemeka Onyemauwa, Blessing Ukeje, Chinwe Eluagu, Olamide Olaosebikan, Benjamin Okoye

**Affiliations:** ^1^National Root Crops Research Institute, Umudike, Nigeria; ^2^International Institute of Tropical Agriculture, Ibadan, Nigeria

**Keywords:** social movements, institutions and governance gender, good practices, end-users perception and processing traits, cassava, plant breeding traits, Nigeria, plant breeding gender

## Abstract

Including gender research in cassava breeding makes it easier for farmers to adopt new varieties that meet the specific needs and preferences of both male and female farmers, leading to increased adoption of new varieties, improved productivity, and better economic outcomes for the entire farming community. Gender was included in 2013 in variety development at the National Root Crops Research Institute (NRCRI), Umudike, Nigeria in response to the dis-adoption of some varieties by farmers who had not been part of varietal development from the start, and in light of social roles which influence the responsibilities, resources and livelihood outcomes of men, women and youths. Gender inclusion has given plant breeders accurate information about the cassava traits preferred by all end-users, not just male farmers. At NRCRI, gender studies intensified in the last 5 years, contributing to the development and release of improved varieties. Quantitative and qualitative research by the gender cross-cutting team modeled trait profiling and consumer preferences, to aid demand-led breeding. Some of the methods were acquired at several trainings on how to quantify qualitative responses for prioritization. Gender research techniques include participatory varietal selection (PVS), participatory plant breeding (PPB), mother-baby trials, focus group discussions (FGD), surveys, value chain mapping, G+ tools, experiments in farmer field schools (FFS), demonstration farms, and tricot. These gave the cross-cutting team a better understanding of gender relations, power, decision-making, ownership and control of resources, and have mitigated operational and field challenges during the surveys. These methods also elicited feedback from end-users that led to better naming of newly released varieties, reflecting perceptions of agronomic performance, and food qualities, which made the varieties easier to identify and remember.

## Introduction

Cassava, an important staple in Nigeria, is widely eaten by people of all ages, and is in great demand in all regions. Cassava is processed into many kinds of foods, which are culturally important for different social groups, across various agro-ecologies.

Gender studies is an essential field of study that examines the social, cultural, and biological differences between men and women, and how these differences impact various aspects of society. One area where gender studies are particularly relevant is in the field of agriculture and food production. As indicated by the Food and Agriculture Organization of the United Nations, “Women are the key actors in processing and food preparation” (FAO, 2011). Therefore, it is crucial for breeders to consider female-preferred traits when developing new agricultural products.

In many cultures, women are primarily responsible for food preparation and processing. This means that the traits they value in agricultural products, such as taste, texture, and ease of preparation, are of utmost importance. Neglecting to consider these preferences can result in a disconnect between the products being developed and the needs and desires of the individuals who are responsible for utilizing them.

Furthermore, gender studies can also shed light on the unequal distribution of labor and resources within the agricultural sector. According to the World Bank, women make up 43% of the agricultural labor force in developing countries, yet they often have limited access to resources such as land, credit, and technology (World Bank, 2009). Understanding these disparities is crucial for developing more equitable and sustainable agricultural practice.

This diversity in cuisine influences varietal adoption. When a recently released variety lacks the traits of interest to end-users, they may try the new cassava, and then abandon it. For instance, women who process cassava into yellow *gari* in Benue State were early adopters in 2011 of yellow, cassava, biofortified with vitamin A. But in Oyo State, the local market demanded white *gari*, so yellow cassava was only adopted where yellow *gari* was made for sale in cities like Lagos. *Gari* (or cassava semolina) is a gelatinized, fine to coarse granular flour made from grated and fermented cassava roots (Teeken et al., 2021). Women adopters who did adopt yellow cassava said that it needed improvements such as roots that would not rot after 12 months in the ground, dryer roots (less water content) and cassava that would retain more carotenoid after processing into *gari*. The women processors are able to assess the carotenoid retention by observing the intensity of the orange color in the gari (Ilona et al., 2017; Olaosebikan et al., 2019), which informed breeders as they developed the second and third waves of biofortified cassava, released in 2014 and 2021.

Through conventional breeding, these improved, biofortified, third wave cassava now have more dry matter, mealier texture, and the roots can be left unharvested for longer without rotting. If communities lose access to the planting material of improved varieties, farmers return to planting local cultivars, which are readily available. Even if end-users like the new varieties, they are often dis-adopted because farmers do not always save stems, and the planting material may become locally unavailable (IITA, 2015; Bentley et al., 2017). Unlike grain crops, cassava is reproduced vegetatively, with stems, which are perishable, so it is difficult for farmers with little land to conserve the varieties. Women have limited access to land, so they grow less cassava and process more of it (some of which they buy). Women tend to run out of stems more often. Dis-adoption of new varieties, either because farmers do not like the new varieties, or cannot get the planting material, limits the achievement of key breeding objectives, such as improving yields and livelihoods.

By 2015, evidence documented by social scientists at NRCRI and partner institutions, showed that men and women may prioritize different cassava traits (Bentley et al., 2017; Teeken et al., 2018). Some specific traits are preferred by the men and women who perform particular tasks in the cassava value chain. Because consumers rejected varieties lacking preferred traits, cassava breeders recognized the need for information to design varieties that meet the demand of producers, marketers, processors and consumers. The researchers also realized that this information should capture the different needs of women and men.

Age, class, ethnicity and gender affect access to resources (Madu et al., 2021a). Gender differences on their own are too simplistic to explain all of varietal choice and adoption. Categorizing users to include gender while taking into account other important social characteristics is vital. When selecting participants in our studies, the team used a task group focus. We define a “task group” as a set of people with hands-on experience in cassava farming, marketing and processing. We never used sex as a direct criterion for selecting informants; instead we identified the different tasks performed by men, women, or both in cassava production and processing and marketing, and then we selected participants who were well-informed on various tasks. These task groups usually include men and women who perform the tasks locally ascribed to their sex. We also considered intersectionality, where participants come from different age groups, or ethnic groups, where each one might represent a different mode of production (more mechanization vs. less, or hired vs. household labor).

This task group approach focuses on who does what along the value-chain and allows for a close integration with participatory trials, post-harvest processing, and breeding, which are all connected to specific tasks. People can simultaneously belong to different task groups and the extent to which men and women do certain tasks, and belong to one group or another are informative about gender roles, norms and possibilities within farming, processing and selling. The task group approach fits in the “social targeting and demand analysis” breeding stage, but could also be used within participatory breeding strategies (generation of new varieties). Some cassava processing activities like grating may have mixed a gender composition. Toasting *gari* and preparing *eba*/*fufu*/*akpu* are mainly by females, but male youths may help their mothers. *Fufu* is a traditional fermented food product in southern, western and eastern Nigeria and other parts of West Africa (Rosales-Soto et al., 2016; Chijioke et al., 2020), usually described as a “wet paste food product” ranking second after *gari* as a food product from cassava (http://www.cassavabiz.org/). In some regions men do the jacking: to press grated mash with a hydraulic truck jack.

### Context

Most Nigerians rely on cassava, yet the country never has enough of it and the prices of derived products such as *gari, fufu*, and cassava flour, keeps increasing (Ekott, 2021). Globally, Nigeria ranks first in cassava output (60 million tons) and land devoted to the crop (7.7 million hectares) but 66th in productivity (7.75t/ha). In Nigeria, about 90% of cassava roots are processed into food (as opposed to alcohol, for instance). Seventy percent of the cassava produced is made into *gari*, and almost all of the rest is prepared into foods like *elubo* or *lafun, fufu*, or *abacha* (Otunba-Payne, 2020). *Gari* is preferred over *fufu*, with its short shelf life and tedious processing methods (Chijioke et al., 2020).

More than 500 million people in Africa eat cassava foods daily (Bakum, 2020). Cassava is more resilient than maize and wheat to climate change and is expected to play an important role in ensuring food security in Africa in the decades ahead. Some cassava varieties can be eaten fresh, and some derived products (e.g., starch) are crucial as industrial raw material. The potential demand for cassava is high, in part because the diverse ethnic groups of Nigeria make it into so many kinds of foods. Preferences for food characteristics can be influenced by socio-economic status, household size, culture, and health perceptions (Bello et al., 2020). Some cassava projects like NextGen and RTBfoods developed research methods to identify traits preferred by men and women farmers, processors and end-users for specific food products. These make cassava breeding demand-driven, gender-responsive and inclusive; to enhance the probability of adoption of newly released varieties (https://www.nextgencassava.org/).

These projects have used different approaches to identify trait preferences, such as direct ranking (Abeyasekera et al., 2002; Dao et al., 2015; Teeken et al., 2018), or choice experiments (Asrat et al., 2010; Blazy et al., 2011; Acheampong et al., 2018). Some trait preference studies have addressed social differences among cassava producers, processors and end-users (Chijioke et al., 2020; Ndjouenkeu et al., 2020; Teeken et al., 2020, 2021; Forsythe et al., 2021; Madu et al., 2021a,b,c,d). Bechoff et al. (2018) indicated a dearth of information on gender-specific crop trait preferences, because they are rarely considered in breeding programs. Teeken et al. (2021) noted that various studies have identified gendered trait preferences, without analyzing how they interact with household characteristics, and geography. The complexities involved in processing major cassava products (*gari* and *fufu*), mainly by women, calls for introducing a crosscutting team and gender mainstreaming, to breed varieties that meet end-users' needs for *gari* and *fufu* (Chijioke et al., 2020). Success stories are needed to explain how newly released varieties incorporated end-users' preferences.

## Analysis

### Multidisciplinary approach for inclusive cassava breeding

Gender studies at NRCRI Umudike started around 2015 for cassava, introduced by the NextGen Breeding Project, a multidisciplinary collaboration among gender experts, breeders, food scientists, social scientists, extensionists, statisticians, and economists during field trials. In the beginning, gender experts and social scientists were saddled with the task of identifying stages win the breeding cycle where gender approaches help to make breeding inclusive and reduce gender-blind trait prioritization.

This was followed by a mixed methods approach involving surveys, feedback exercises and a gender- intentional method for selecting participants with purposive sampling. The baseline and monitoring surveys, stakeholder interviews, and focus group discussions (FGD) targeted all actors within the cassava value chain (producers, processors, marketers and end-users). The extension specialists within the institute and national extension agents worked hand-in-hand when visiting or selecting communities, and for community engagements before the interviews. The multidisciplinary team validated the survey instruments before fieldwork. Field data were cleaned, stored and organized for analyses. The breeders managed samples (leaves and roots) from the field, while the food scientists performed laboratory testing and sensory panel testing of food products (Forsythe et al., 2021). In the early years, breeders only relied on the laboratory, trained panelists and sensory testing for food product evaluations, which were useful, but rarely depicted real-life experience. Recent projects such as NextGen Cassava Breeding, RTBfoods and 1000FARMS, have piloted methods for consumer testing, such as check-all-that-apply (CATA), just-about-right (JAR) and triadic comparison of technologies (tricot) (van Etten et al., 2019a, see Table 1).

**Table 1:** Methods and approaches.

**Activities/timeline**	**Description**
Participatory Varietal Selection (PVS)-−2020–2022, still ongoing, scaling to other crops in Nigeria	The Triadic Comparison of Technologies (tricot) method was managed by farmers in their own fields. Farmers were given 3 varieties of cassava and a protocol to set up small plot trials that were easy to manage. Farmers were trained as citizen scientists to collect data electronically and give feedback to the researcher. Farmers reported their choices of the agronomic and food quality traits of the varieties. Important criteria for adoption, which are easily overlooked at researcher-managed trials, are accounted for by the men, women and youths participating as citizen scientists (van Etten and Steinke, 2021). This approach is now being adopted by the variety release committee in Nigeria. The team (breeder, gender expert, social scientist and food scientist) participated in anchoring tricot trials for a PhD students' work in breeding in (2020–2021). All the disciplines worked together harmoniously bringing in their expertise
Participatory Plant Breeding (PPB)	From the PVS, demand-driven research and decision-making is enhanced to facilitate screening of promising genotypes to advanced stages of plant breeding, resulting in the release. The farmers, especially participants in the mother-baby and tricot trials, assessed the traits in the field
Mother-Baby Trials (2017–2019)	NRCRI and IITA set up mother-baby trials in South-East and South-West Regions of Nigeria to engage women and men small holders and processors from 2017–2019 to enhance targeted and demand-driven breeding (Teeken, 2019). Preferred varieties included NextGen clones (Teeken et al., 2021)
Citizen science involving mass volunteer participation in the research (2017–2022)	Using the RTBfoods method, interviews with women leaders and community leaders, focus-group discussions with participants in the value chain. Marketers study (especially SoK, gendered food mapping, and consumer testing). Elicited trait preferences from many and diverse farmers
Experimenting farmers formally organized in groups or committees or networks to contribute to the breeding	Female farmer-processors who participated in the tricot and mother-baby trials successfully organized themselves into groups to enhance access to information on new technology
Farmers evaluating and selecting segregating materials	Similar to PPB. Successfully conducted with participants in the RTBfoods, NextGen projects. Farmers participated in the pair-wise ranking of the genotypes at harvest and processing stages (Forsythe et al., 2021)
Social survey research	Life history analyses conducted among cassava farmers in 2018 in Imo and Osun states. Adapted GENNOVATE life history interview guide to obtain qualitative information on the importance of cassava livelihood and the social categories of actors. Gender roles, trait prioritization, asset management, independence, aspiration to power and freedom, were elicited and disaggregated by gender (Olaosebikan et al., 2021). Social segmentation was gender disaggregated (with key informant interviews, FGDs) in terms of livelihoods, wealth categories (Forsythe et al., 2021). RHoMIS–Rural household multi-indicator survey (Hammond et al., 2017), tricot (de Souza et al., 2024), poverty probability index (PPI) and 1,000 minds surveys were conducted between 2020 and 2021 to gain in-depth understanding of preferred traits by geographical location (Balogun et al., 2021)
Value Chain Analysis or Mapping (2021)	The value chain mapping for *gari* showed percentage contribution to the different nodes by production, processing, wholesale, retail, consumption (fresh and processed forms), all disaggregated by gender
Study of trait preferences	Trait preferences disaggregated by gender were elicited in RTBfoods, NextGen, and tricot-Rhomis projects by NRCRI and IITA from 2017 to 2023 for targeted breeding and demand-driven research (Madu et al., 2021a; de Sousa et al., 2023)
Use of G+ Tools for consumer or product profile assessments	G+ tools were applied for product profiles (*gari* and *fufu*). Possible harm and positive benefits were highlighted for traits at the production, processing, and consumption stages for both *gari* and *fufu* (Madu et al., 2021a,b)
Farmer-managed or small-scale, seed production	In their study on Differentials in the Cassava Seed System among Entrepreneurs in Southern Nigeria: A Gender Situation Analyses, Madu et al. (2022) highlighted issues on gender roles, seed drivers, seed flow, profitability, constraints militating against the cassava seed system
Farmer field school experiments or demonstrations	The adopted village project and school outreach program is a pilot project designed to facilitate the transfer of agricultural technologies developed by NRCRI through participatory demonstration plots in schools and communities. Both genders and youths are involved in farming activities in primary and secondary schools and rural communities (NRCRI, 2012).
Tricot Model	Cassava farmers (male, female and youths) managed and assessed 3 pre- released varieties of cassava throughout the value chain to determine which variety is best, intermediate and worst, based on preferred traits. This model informs the breeders and the variety release committee of the desired traits by gender (de Souza et al., 2024). The trial could not always be implemented for a year, as some volunteers were migrants who rent land. Herders destroyed some farms

These methods were used to document trait preferences for roots, *gari* and *fufu* among men, women and youths in the different value chain segments. The G+ tool was used to document trait trade-offs (Forsythe et al., 2021; Polar et al., 2022) to corroborate priority ranks assigned to the traits. Going beyond sensory traits (e.g., taste), our studies also identified the influence of socio-demographic characteristics, location and food habits on the preferred quality traits of *gari* and *eba* in Nigeria (Teeken et al., 2021; Olaosebikan et al., 2023). There is now a well-established and cordial relationship among the economists, data curators, application developers, data analysts, statisticians, breeders, social scientists and food scientists as they work together to analyze traits and present them in an easy- to-understand format that can be incorporated into the breeding programs.

### How attention to gender influenced cassava breeding in Nigeria

The socio-economic team brought to breeders' attention issues raised in previous adoption studies, e.g., the social dynamics, and cultural norms that influence the assignment of responsibilities, and scarce resources and the livelihood outcomes of men, women and youths (Quisumbing et al., 2014; Orr et al., 2018; Polar et al., 2021a,b). The responses of men and women in several surveys indicated significant complementary and some dissimilarity in trait preferences and varieties adopted and dis-adopted. Going forward, NRCRI participated in trainings and projects such as Gender-Responsive Equipped Agricultural Transformation (GREAT), RTBfoods (for four product food profiles; boiled yam, pounded yam, *fufu*, and *gari*), and NextGen Project (including tricot, Rhomis, 1000 minds surveys). These projects aimed to package gendered consumer preferences into insights that breeders could use. This increased the efficacy of selection aimed at increased adoption of improved root and tuber crop (RTC) varieties in Africa.

## Methods and approaches

### Impact of multidisciplinary training and approaches in cassava development

The GREAT training in 2017 provided hands-on practical training on how to mainstream gender into breeding. It also strengthened the relationships among disciplines, enabling them to work together as a team. Most teams during the GREAT training comprised three individuals who could have been a breeder, a gender specialist, a food scientist, or a social scientist. The team relationships fostered and were scaled into projects like RTBfoods, which further showcased the benefits of achieving objectives as an inter-disciplinary team, especially the deep understanding of traits preferred by end-users (Forsythe et al., 2021) and how this knowledge could be translated into markers and biophysical characteristics for use by breeders and food scientists. These exercises within the RTBfoods and Nextgen projects have provided knowledge of new trait categories for the cassava ontology database, cassava base and breed base (Agbona et al., 2022).

**Figure 1:**
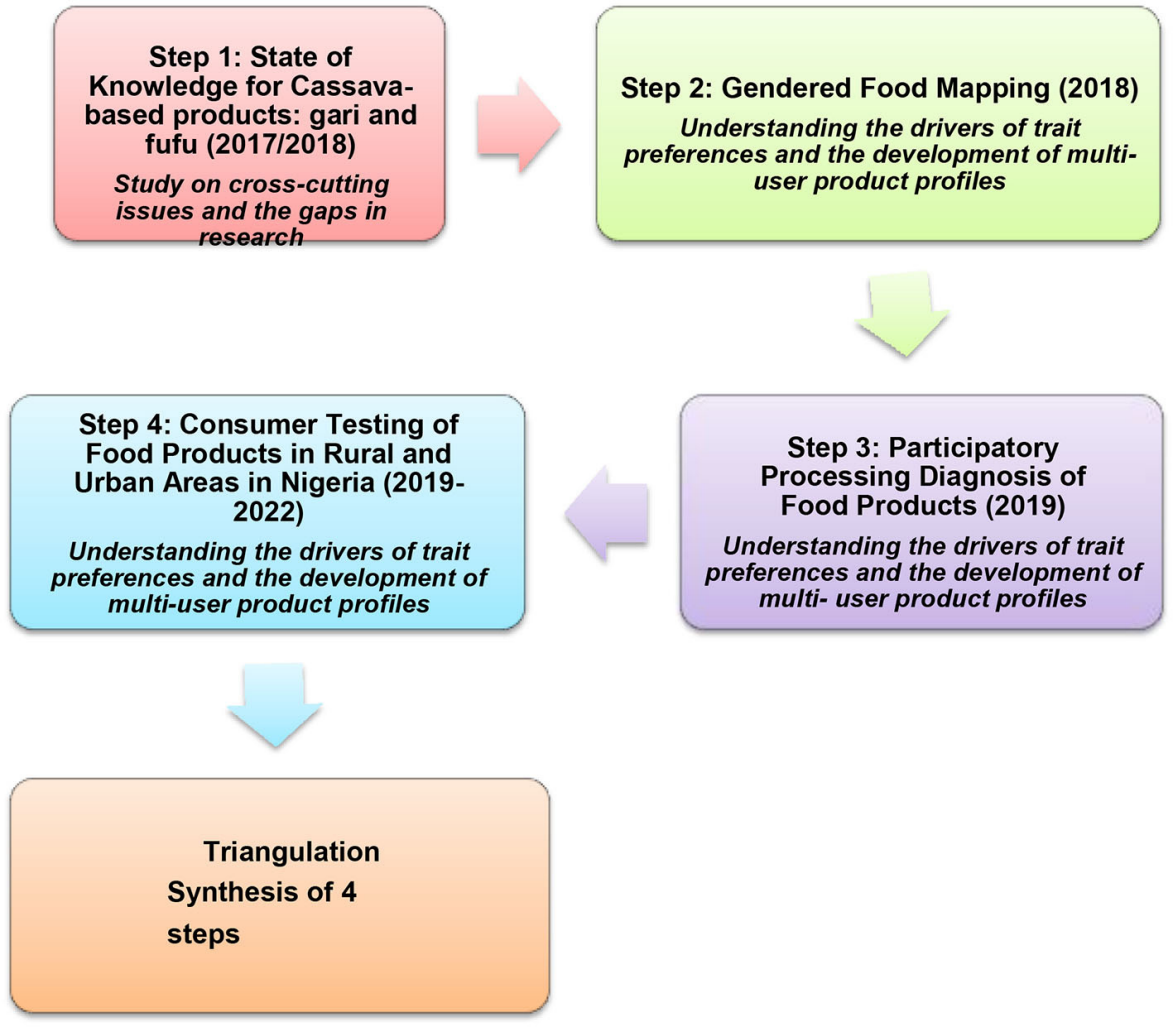
Timeline and steps in the method and integrated field approach to enhance variety development.

Figure 1 shows five main stages in the development of gender-responsive breeding at NRCRI following the RTBfoods method. The RTBfoods project from 2017 to 2022 adopted an integrated field approach for developing food product profiles for *gari* and *fufu* along gender lines (Forsythe et al., 2021). Step 1 provides the scope of the study and identifies the gaps in research (SOK-State of Knowledge) (2017 and 2018). Step 2, in 2018, studied ranked quality characteristics among actors playing different roles on the food chain and described in-depth context of the research for *gari*-*eba* (Madu et al., 2021a). For Step 3, champion processors were engaged in 2019 for a deeper analysis of *gari*-*eba* and *fufu* quality characteristics (Madu et al., 2021b,e; Teeken et al., 2021).

Processors are an important interface, linking agriculture (knowledge of raw materials) with the market and end-users (knowledge of consumers' expectations). Step 4, from 2019 to 2022, provides robust data on final product preferences among a diverse set of end-users (Madu et al., 2021c,d). Developing questionnaires and implementing Step 4 benefited from the experience of Step 3. A gender specialist, a market economist, a food scientist, and extension specialists were among the Gender Team that conducted the fieldwork during all four steps. The team participated in several gender workshops and trainings organized by NextGen and RTBfoods projects covering fieldwork and social surveys to integrate gender. During fieldwork, several specialists were on hand to respond to agronomic, processing and use issues raised by the respondents.

Results from the different steps were then triangulated to prioritize traits for the Food Product Profiles, so that traits preferred by end-users could guide breeders. This is a bottom-up approach rather than the conventional top-down approach (breeders' product profile) used by breeders. The integrated method enables a deep understanding of the quality characteristics, translating tacit knowledge into data that can be further investigated by scientists (Polanyi, 1966, cited in Forsythe et al., 2021). Triangulation (Figure 1) is a synthesis of 4 steps (Forsythe et al., 2021) of research with value chain actors. The lab- based sensory evaluation corroborated the results from Steps 3 and 4, using scientific and industry standard methods.

In addition to the four Step methods for defining Food Product Profiles, the team also applied the G+Product (Table 1) Profile Query Tool. The G+ tools helped gender researchers and breeders make joint, evidence-based decisions about gender differences and trade-offs when deciding to include gendered and food product traits such as color, *gari* and *fufu* yield, going beyond agronomic traits previously emphasized by breeders. The G+ tool (Table 1) was applied to the products, to describe the characteristics that can determine whether or not a variety is likely to be grown and eaten as a cassava food product (Polar et al., 2022). Characteristics were assessed using the positive benefit and “do no harm” tool to decide gender impact scores for aspects such as drudgery reduction, use of hired labor, access to essential inputs, product quality and quantity, and positive valuation of traits by men and women. Advantages of this tool include: easy to comprehend and use, application of both quantitative and qualitative responses to determine trade-offs and prioritize important traits.

The quantitative and qualitative surveys combined the following tools: rural household multi-indicator survey (Rhomis), poverty probability questionnaire, and 1000minds surveys (social survey research, Table 1). For instance, the 1000minds-Rhomis results reinforce the importance of recognizing social differences among men and women, and how individual and household characteristics interact to influence trait preference variability. This information can inform trait prioritization and guide development of breeding products that have higher social impact, serve the more vulnerable and align with development goals (Teeken et al., 2021; see Balogun et al., 2021).

### How the breeding changed as a result of the gender research

To meet end-users' requirements, breeders must understand the priorities, and constraints that women and men along the value chain assign to crop and animal products. Disaggregating trait preferences by sex will enhance the adoption of innovations among farmers, thereby, strengthening food and nutrition security (Tufan et al., 2018).

Research toward this end started with on-farm trials (PVS, mother-baby trials) to assess which traits end-users value for cassava and its products, and if users were willing to pay for any of these attributes (e.g., disease resistance and nutrient-rich varieties). Project researchers evaluated the trials with the farmer-processors as the crop grew, combining this information with social science tools to inquire into the aspirations and possibilities of participants. This more accurate qualitative research approach allows participants to correct, and refine their opinions. We subject most of our participatory breeding and survey data to gender and content analysis to extensively inform breeding.

Life history research (social survey research) was done with the participants who participated in the PVS and mother-baby trials. The study found that the impact on cassava-related livelihoods for men and women is greater when complemented with capacity building need by each task group (Olaosebikan et al., 2021). The use of household labor for weeding poses a challenge to women farmers, who need varieties that suppress weeds. Impacts on cassava-related livelihoods go beyond food and economic benefits, extending to women's aspirations to the power and freedom to make major life decisions.

The PVS and mother-baby trials (Table 1) aimed at enhancing the adoption of genotypes for release. Highly motivated participants maintained quality trials. In the task group approach, more experienced and knowledgeable participants were selected which gave insights to traits that breeders can work on. Results show that NextGen varieties are competing favorably with common farmer varieties. Acknowledging farmers as research partners encouraged their interest, dedication and curiosity. The use of social science research methods helped to facilitate participatory plant breeding.

During research, the team gained a better understanding that gender is shaped and articulated by the different social identities to which people belong. Currently, we are piloting the tricot citizen-science approach (Table 1-Tricot model) that involves men and women purposively selected to evaluate new varieties. This trial helps to target certain varieties to specific groups (farmers, processors etc.), and identifies who buys which cassava-based products, and who has access to resources. Other issues include: power, decision-making, diversity and communication, and the changes that would enhance gender equality and increase access to opportunities and benefits from production, processing and marketing. We included participants from different local groups and socio-economic classes. Each group represented a different mode of production (more or less mechanized and using different labor sources) to also target drudgery.

### Breeding outcomes and impacts

Previous to including gender analysis in the research, the breeders felt that social science was unimportant, saying, “(social scientists) ask too many questions and waste too much time in the field.” This attitude prevailed until the start of the NextGen project, which came with the gender component, and an NRCRI Multi-disciplinary Breeding Team was created that engaged social scientists in field trials and all nodes of the value chain. The Team participated effectively in all the methods and approaches listed in Table 1, following the procedure in Figure 1. Qualitative and quantitative data were taken at the pre-harvest stage, with farmer participation in mother-baby and tricot trials. The social scientists learned on the job and helped collect agronomic data. Eliciting qualitative data from the participants revealed the social reasons behind why they preferred particular agronomic traits. This was also followed with post-harvest activities, processing and consumption (led by a food scientist). The breeders saw the need to understand trait preferences and ranking at all nodes of the value chain, instead of merely gathering field data on yield and morphological characteristics (such as stem diameter). Some varieties preferred for traits like yield, disease resistance, or even dry matter (processing), were dropped during sensory (taste) tests. For example, a particular farmer who assigned a low score to one variety during a field assessment later gave it a high ranking at the final product stage; she said “*I wish I could apologize to the cassava variety for scoring it low at field assessment, because of its architectural formation*.” The G+ tool was perfectly suited to reconciling such differences, so traits could be prioritized, allowing breeders to concentrate on demand-driven traits.

Our studies have shown clear evidence in produce (root) and product (*gari* and *fufu*) trait preferences among men, women and youths within the different value chain segments; this came out more clearly in the task groups, where gender interacted with other social identifiers. This also is supported by trait trade-offs (with the G+ tool) (Polar et al., 2022), to corroborate priority ranks assigned to the traits. Our studies also identified the influence of socio-demographics and consumption patterns on the preferred quality traits (Teeken et al., 2021). Our finding (Chijioke et al., 2020), reveals a link between the preferred root quality traits (root size, heaviness, appearance), processing traits (ease of peeling, texture and high retting ability) and overall quality of cooked *fufu*. These quality attributes provide insight to cassava breeders as they redirect breeding activities (Table 2).

**Table 2:** Changes in breeding initiatives emanating from gender inclusivity.

Definition of market(s) or consumers to be targeted	Gender analysis showed that men and women have different roles in cassava value chains that affect their trait preferences. Men and women were involved in each node of the value chain. These include the direct players (producers, processors, end-users etc.), and the indirect players who facilitate the chain (transporters, loaders/off-loaders, input dealers, credit agencies etc.). Product testing now extends beyond the four walls of the laboratory to end-users in villages, towns and cities. In addition to gender disaggregation and integration, the field team is now intentional and sensitive to enabling equity, e.g., purposively sampling diverse ethnic groups during surveys. A value chain approach is an effective way to make sure stakeholders other than male farmers and female food vendors have a say in varieties with the right qualities
Breeding objectives	Setting objectives with stakeholders was valuable for organizing product profile and advancement meetings with the inter-disciplinary team, to validate the continued inclusion of existing traits such as yield, and disease resistance. Social and gender analysis showed the importance to breeding objectives of prioritizing gender and task traits such as color, easy to peel, increased dry matter content. There is a planned stakeholder's validation meeting to discuss traits needed to boost cassava's resilience in the face of climate change and conflicts
Breeding strategy or methodology	Screening promising genotypes with key traits. Multi-disciplinary meetings facilitate in-depth understanding of traits, a robust ontology database (https://www.cassavabase.org/tools/onto/) and new learning. The breeding method has changed because of the knowledge that preferences vary for men and women. Gender is now taken into account in breeding efforts
Criteria used to evaluate the importance of different traits	Gender analysis broadened the Team's criteria for evaluating traits. Beyond food security, a gender-responsive poverty index (PPA) and industrial criteria to meet specific needs have been incorporated. Stress-resilient traits should be added next. Multi-disciplinary criteria are used to evaluate traits. Farmers, processors, marketers and consumer preferred traits are triangulated with biophysical and functional analysis for a deeper understanding of traits
Relative importance or weight given to different traits	Gender analysis using the G+ tool together with the assigning of economic values to traits has facilitated analysis of trade-offs and weighting traits for their prioritization. Using the G+ tool to prioritize trait preferences and assign gender impact scores improved the prioritization of weighted traits
The traits given priority by the breeding	Including gender analysis in the multidisciplinary approach from the start of breeding made it easier for the Team to identify traits preferred by the end-users, with the involvement of the consumers (gender disaggregated). Breeding for the end-users facilitated adoption of new varieties by all stakeholders. Involving women allowed discovering crucial traits such as final product weight, retting ability, color, texture, and in-ground storability
Methods for evaluating new material on- station or on- farm	The tricot approach (van Etten et al., 2019b) has been adopted for on-farm trials by the Variety Release Committee in Nigeria, shortening the time frame in the breeding cycle (the Nigerian variety release system is one of the lengthiest in Africa). Validate and disseminate new technologies with ease, collaborating with many participants under diverse conditions (van Etten and Steinke, 2021). Results are quick, and local systems are strengthened because more choices are available. Variety selection is influenced by gender
Choice of materials to advance to the next stage of breeding	Tricot gives multiple opportunities to include many genotypes and many farmers. In Nigeria, 30 genotypes were tested with 320 farmers in 2 regions in the first phase of the trial. Based on farmers' evaluations, some clones were dropped because they lacked priority traits. We were then left with 28 genotypes for the next level of evaluation. It is easy and logical to have the farmers help decide which varieties to drop, and to explain why. This approach captures gendered interests
Seed multiplication and dissemination	Use of certified seed companies like Umudike Seeds Ltd, Village Seed Entrepreneurs (CSEs), and different research programs in NRCRI, Umudike. Building an Economically Sustainable Integrated Cassava seed Systems II (BASICSII) project ensures that women are effectively involved as CSEs to encourage use of certified seeds. This helps to ascertain the quality of seeds been disseminated and to keep track of them. The CSEs help in rapid dissemination of planting material (Bentley et al., 2020)
Tricot Model	The tricot approach provides a rating scale from 1 to 3. Choosing between best and the worst is easier and gives an unbiased trait assessment. End-users' cognitive load is less: they deal with 3 different varieties per assessment (faster interviews, so less fatigue), more accurate data. More varieties can be evaluated at once. More farmers are involved without increasing the time spent on consumer testing. Plot trial is manageable for both men and women. Direct and easy data collection (ODK), bar-coding-unique identifier, and automated archiving & analysis in ClimMob cloud server. Structural integration of gender and socio-economic variables. Allows the integration of just about right (JAR) and check all that apply (CATA) and Overall Liking (OL). Transparent and easy monitoring of data collection can be shared with project implementers, partners and donors. ClimMob ODK-GPS enabled collection is more efficient in locations with excellent internet connectivity. This approach allows more participants to be engaged in on- farm trials at more locations. Engaging farmers in research gives them ownership over it, makes products more acceptable, and promotes extension services which are a major driver of adoption of new varieties
Varietal naming and product launch (2021)	Feedback from end-users have informed the naming of new varieties from the usual numbered or scientific coding, names such as TMS30572 to names suggested by farmers depicting how the varieties are perceived such as Ayaya (CR36-5) which in local dialect means beautiful, Fine face (IA980505), Game changer (TMS13F1160P0004?) Obasanjo2 (TMS13F1343P0022), Sunshine (TMS/IBA070593), Hope (NR130124), Dixon (TMS/IBA90581). These names are easier to remember, especially for women, facilitating their access to cassava seed, knowing the exact seed they want
Participants and respondent designation changed	Cassava value chain actors have changed from mere participants and respondents to citizen science partners in research, with signed agreements between the research institute and partners. They are research partners providing needed information, evaluation and guidance to the breeders. Participating men and women become role models in their communities

As a result of the successful gender studies, cassava breeding initiatives have recently prioritized two more food product profiles, focused on food security, and on improved nutritional quality and industrial needs. These include (1) processed foods, which extend the short shelf life of the cassava root, and (2) fresh cassava for eating after boiling at times of the year when yam is expensive or unavailable (Olaosebikan et al., 2023).

## Discussion

### Good practices

The gender findings demonstrate that it is imperative to introduce a multidisciplinary, multi-stakeholder approach in crop breeding, and to include the science behind food processing, marketing and consumption. This will help to breed varieties that meet end-users' needs and enhance the adoption of these new varieties. Female-preferred traits should be of utmost importance to the breeder, since women are the key actors in processing and food preparation. Findings identified the importance of demand-led gender-responsive breeding, trade-offs between traits as a guide for breeders.

Our successes in the research were achieved as a result of imbibing team spirit in our work, where we assist one another to make sure that tasks and timelines are completed. We organize planning meetings, team and capacity building, feedbacks and appraisal exercises, which enable us to plan tasks and set timelines to ensure we meet our deliverables. The team members are always ready to assist each other in the field. This includes mentoring by experienced leaders to teach resources (effective gender budgeting), and mobilization when needed to ensure smooth delivery of assigned tasks. Communication was also effective, making use of social media platforms such as WhatsApp, in-person and virtual meetings. With minor hitches, we bridged some of these gaps by carrying each other along, sharing ideas, and engaging more respondents, especially women who dominate the cassava value chain in Nigeria. Capacities of researchers were built on gender mainstreaming; making them more aware and gender-sensitive going forward in future projects and programs. The synergy among the multi- disciplinary team has enhanced effective breeding programs.

Outcome of lessons from learning visits facilitated by gender experts for Nigeria include: the importance of verbatim notes, and quotes; using words as the community expresses them; quantifying qualitative statements; using pictures in the sand, bottles, to compare sizing, shape etc. Pairwise ranking was more reliable in terms of understanding priorities than ranking, but is still not perfect; the G+ tool made better analyses. Some respondents are less responsive and have more trouble with the questions. The interview followed with “step by step” questions, which helped the respondents flow along Dufour et al. (2019). At the start of fieldwork, debriefing after each day's work follows with the whole team to discuss how questions should be asked, challenges and modifications. Roles for facilitation and note- taking were clearly defined. Interaction and support by all team members empowered one another. Showing organization and a friendly disposition, along with continual engagement with the respondents, was necessary. Scheduling of interviews and meetings was essential, especially for women (to enable non-interference with religious and social engagements). Expectations with the mobilizer were discussed and expectations were clarified at start of the interview. Piloting the tools is vital and each team member needs to have the same interpretation of the questions.

Women tend to answer questions clearly, while men always seem to be in a hurry. There is need for gender-inclusive methods, integrating activities for men and women for targeted breeding objectives. An enabling environment should be provided for men and women to air their voices, freedom to discuss, argue and reach a consensus in FGDs and other meetings. Opinions become more transparent as women and men are involved in decision-making and managing trials (mother-baby and tricot trials). Ways in which gender norms shape their preferences for varieties and traits can therefore be elicited. Sometimes women need a space, without men, to be able to express themselves better. Key informants, such as role models, community and women leaders helped to explain ambiguous responses from some individuals.

Sometimes we experience some hitches when respondents (especially the men) are impatient to spend extra time as we elicit responses. As we probe to shed light on an earlier response, the farmer sometimes become tense and responds “I thought you asked me this question before.” The respondents, especially the women, are usually engaged in many activities on their farms and at home. Sometimes they combine farming with petty trading to make ends meet, so they find it difficult to settle down in one place to answer questions for long. An example, one of our farmers also owns a restaurant. During our interview, she couldn't concentrate to respond to questions because of customers coming in for meals, so we (the socio-economist and the food scientist) resorted to washing dishes to ease some of her burdens, giving her the time to answer our questions. We discovered that when we combined male and female farmers in the FGDs, we tended not to get the desired results, because the women usually remained silent and quietly agreed with the men, probably because of power play, and the local norm that women shouldn't speak up in the presence of the men. So we learned to separate the communities into male and female groups, and the women would speak freely.

Key informants selected for our interviews are usually indigenes, local leaders, women leaders and others who are knowledgeable about the community. This yields correct information about the community under study. We select knowledgeable farmers for the studies, irrespective of their social class. When we start to work in a community, we usually go through the village heads to explain our mission and to get to know them. When we start the survey proper, we introduce ourselves to the community, explain the purpose of our visit and read out the consent forms before inviting people to sign them, to indicate their willingness to participate.

In most of the study communities, many farmers are attracted to our style of work, especially in the Tricot and RTBfoods projects, where the farmers go home with all the farm produce after our data collection and also take home some small gifts that we give them to acknowledge the time they have given the project. This motivates farmers to enthusiastically join the project. At the end of the study, most farmers will have access to planting materials for the next season and also for sale. They also get a lot of *gari* after processing cassava as part of the mother-baby trial. On several occasions farmers requested more studies in their communities, indicating interest in our work because it addressed their demands. The farmers who participated in our projects now stand out in their communities and have become role models to others who look forward to such opportunities in future. Listen to the words of four participating farmers in a Nextgen video (NextGen, 2022).

The tricot feedback initiative created another platform for participants to interact as citizen science partners, and it provided suggestions to improve the project. This facilitated co-learning, cooperation, result validation and accuracy, and fostered motivation by presenting research results back to citizen scientists about experiences with the tricot evaluation, which helped to improve the next tricot initiative.

### Lessons learned

The team used participatory gender product profiling trials (see methods and Approaches, and Table 1) across Nigeria to assess popular local and improved varieties and to evaluate traits, including taste and suitability for food preparation. Robust interdisciplinary methods included economics, and qualitative and quantitative tools to capture how farmers, traders, processors, and consumers described high-quality food products in their own words. Respondents were selected to reflect the diverse social makeup of a community. Outcomes include success stories behind on-farm trials and social surveys where traits were prioritized, in order to guide breeders. The study has shown the importance of integrating gender in crop breeding with the use of innovative techniques (tricot, G+ tool etc.) to elicit data from respondents. There are now cassava varieties in the breeding pipeline based on these gender studies. We are currently undertaking a benchmark survey on the adoption of disease-resistant and nutrient-enhanced cassava varieties, including the awareness of women, men, and youths, gender roles in the cassava value chain and factors that encourage or discourage uptake by women and the poor.

## Data availability statement

The original contributions presented in the study are included in the article/supplementary material, further inquiries can be directed to the corresponding author.

## Ethics statement

The studies involving humans were approved by the National Root Crops Research Institute that has the mandate for Root and Tuber Crops Research in Nigeria. The studies were conducted in accordance with the local legislation and institutional requirements. The participants provided their written informed consent to participate in this study. Written informed consent was obtained from the individual(s) for the publication of any potentially identifiable images or data included in this article.

## Author contributions

TM and BO contributed most to the work including writing of the manuscript. All authors made significant contribution to the work reported, conception, study design, execution, acquisition of data, analysis, and interpretation, read through the different drafts of the work, and made substantially and critical contributions to the review of the article.
